# Quantum Information Supports Delayed Decisions

**DOI:** 10.3390/e27090894

**Published:** 2025-08-23

**Authors:** Marius Nagy, Naya Nagy

**Affiliations:** 1 College of Computer Engineering and Science, Prince Mohammad Bin Fahd University, Dhahran 34754, Saudi Arabia; 2 College of Computer Science and IT, Imam Abdulrahman Bin Faisal University, Dammam 31441, Saudi Arabia; nmnagy@iau.edu.sa

**Keywords:** query systems, delayed decisions, quantum parallelism, Grover’s algorithm

## Abstract

Chatbots, search engines and Database Query Systems are invaluable sources of information for decision-making processes in the data-driven world in which we live today. In this study, we explore the extent to which classical and Quantum Query Systems can support future decisions, taken at a moment where the query service may be inaccessible and the decision has to be based solely on information collected in the past. We show that encoding information at the quantum level allows Query Systems to support future or delayed decisions. More precisely, Grover’s algorithm can be employed in order to extract the desired answer from a large superposition of question–answer pairs obtained through a single interrogation of the system. The method works best for binary answers and can be applied to queries encompassing hundreds or thousands of questions in one query. Extensions are possible if we allow more than one query to be addressed to the system. By comparison, a classical system would require hundreds or thousands of queries in order to offer the same level of support for delayed decisions.

## 1. Introduction

A Query System can serve as a theoretical model for a range of services or applications, including Database Query Systems, search engines and generative AI chatbots or agents, to name a few. According to current trends [1], continuously improved chatbots are becoming an existential threat to search engines, as people prefer to formulate and refine their searches in a conversational tone, as part of a dialogue, rather than a plain sequence of keywords. In all of these scenarios, the user perceives the system as a “black box” or “oracle” that can receive queries and responds back with an appropriate answer.

Query systems spread over almost all service-providing software and adapt to or are embedded into a variety of architecture. A few overview examples are given below. In enterprise resource planning (ERP) software, proposals for added-on Query Systems with data processing features combine with large-scale existing proprietary software, such as SAP [2]. For researchers, the development of systematic literature reviews is likened to a selective Query System with distributed data [3].

As Query Systems evolve to cover the stringency of modern business dynamics, query formulation evolves as well. The old and ubiquitous Structured Query Language (SQL) [4] becomes dynamically executed [5] and even proliferates into other standardized variants, such as the Graph Query Language (GQL) [6]. Also, depending on the data structure, the query procedure has to adapt. Blockchain data structures, while designed to prevent data tampering, need to be queried to check or retrieve information. Blockchain queries often trawl sequentially through the blockchain to find information. Therefore, attention has been given to improve query retrieval time by multi-level distributed query [7] or by enhancing the block chain structure with off-chain extensions which improve query efficiency [8].

In terms of architectures that contain data to be queried, the Internet of Things architectures pose new challenges to data querying solutions, as spacial data is located either in devices or cloud servers. As such, the privacy of Query Systems becomes an issue [9]. Spatial data query services are part of common applications, such as taxi dispatching applications and eHealth applications [10], where reverse k-nearest neighbour queries are employed [11] and both queries and answers are encrypted [12].

From a brief overview of the query application landscape, it can be seen that exploring Quantum Query Systems is an untrodden path. By definition, a Quantum Query System will have to work in a different environment than the traditional Internet we know today. Quantum Query Systems require the support of a *Quantum Internet*, a qualitatively different infrastructure, with properties and attributes conferred by the laws of quantum mechanics and which are unavailable in classical networks [13]. A Quantum Internet will fundamentally alter the current paradigm of computation and efforts are made to design quantum computers that are efficient [14] and can support scalable distributed computations [15].

Quantum technologies show promising potential in important areas falling under the scope of Query Systems, such as database retrieval or AI-assisted decision-making. Quantum database retrieval systems can harness the principles of quantum mechanics to enhance computational efficiency and processing speed. The most typical example is Grover’s algorithm [16] that offers a quadratic speedup for unstructured database search tasks. A Quantum Query Language (QQL) is developed in [17] using a formalism based on quantum logic, in order to incorporate retrieval search into traditional database query processing. For a good complete reference exploring quantum information retrieval systems in detail, we suggest the book by Massimo Melucci [18].

Quantum approaches are also increasingly being integrated into AI-assisted decision-making processes. Quantum computing can process vast datasets more rapidly than classical systems, which is particularly beneficial for complex decision-making scenarios in fields such as finance, healthcare, and logistics. For example, researchers are exploring quantum algorithms like the Quantum Approximate Optimization Algorithm (QAOA) to optimize AI models, thereby improving decision-making under uncertainty with respect to bias and transparency [19]. Additionally, the combination of quantum computing and AI can facilitate real-time data analysis, enabling more informed and timely decisions in dynamic environments, such as Internet of Things (IoT) applications [20]. Researchers are also investigating quantum computing’s potential to unlock the capabilities of artificial intelligence applied to healthcare’s most complicated problems [21].

Furthermore, practical implementations of current Query Systems do not take into consideration the advantage of unconventional query settings, such as a delayed query answer. While the speed of query answering is indeed an issue in most applications, delayed answering of a query that is not yet defined is an intriguing setting and will certainly find its niche of applications. In this case, the user is interested in retrieving an answer when the Query System is not available. The user did have access to the Query System in the past, but at a time when the question itself was not known. Thus, the setting of the delayed Query System gives the user the capacity to prepare a “quantum offline” client for a future unknown query.

Now, indeed, what Quantum Query Systems can offer as an enhancement to existing Query Systems certainly depends on the availability of quantum devices and networks. The present study explores this unconventional capacity of quantum settings to add new features to query services.

Many algorithms, including quantum algorithms, can also be analyzed in a Query System model, where the complexity of the algorithm is measured in terms of the number of queries addressed to the black box it uses. In the case of quantum algorithms, the oracle is endowed with quantum properties, allowing it to handle multiple questions from the user in a single query in the form of a quantum superposition state. In truth, some of the best-known quantum algorithms to date can be expressed in this framework of a Query System employing a black box that can answer queries formulated in a certain way: Simon’s algorithm [22], Grover’s algorithm [16], and the period-finding subroutine used in Shor’s factorization algorithm [23].

In this manuscript, the focus is not, however, on classical or quantum complexities of problems that can be formulated in the black box paradigm. Our interest herein lies in exploring the extent to which the quantum computing paradigm can support *delayed decisions* in connection to Quantum Query Systems, a feature that Classical Query Systems do not possess by definition. To be more precise, we refer to the following framework: a single query is made to the Query System containing the user’s question(s), and after a certain time, when the Query System is unavailable, the user has to make a decision based on the available information received as an answer to his query. The limitation to a single query may stem from practical considerations, such as the accessibility of the system to the user, the cost associated with querying the system, etc.

In a classical setting, the user may choose the most important query to submit to the system and hope that the information received in the answer is still relevant by the time the decision is made. In a quantum setting, however, the user may formulate a *composite* query, encompassing many different questions encoded together in a superposition state, and try to extract from the superposition of answers received from the Quantum Query System the one that is most relevant at the time of decision-making. Our detailed theoretical analysis validated by experimental simulations reveals the trade-off between the size of the superposition state encapsulating the set of questions/answers and the accuracy of obtaining the desired answer through repeated applications of Grover’s operator. For binary answers (“Yes/No” questions), a handful of qubits offer enough space for dozens of questions to be encoded in a single query, while allowing the correct answer to be retrieved from the superposition with good probability. Increasing the number of qubits allows an exponential increase in the number of questions that can be squeezed in a single query, but decreases the probability of obtaining the correct answer. Multiple copies of the superposition of answers, obtained by querying the Quantum Query System several times, can boost the probability of success and can also be used as a strategy to generalize the approach to Non-Binary Query Systems, where answers can be encoded on more than one qubit.

Large language models (LLMs) bear some resemblance to our quantum delayed decision scheme, in the sense that it can be incorporated into the workings of the server reading the superposition of questions and providing the superposition of answers. LLMs typically have huge resource requirements (power, training data and time, cooling water), but some lighter versions can be locally downloaded by a user, in which case, the user can access the knowledge provided by the LLM later. Note that in this case, an entire LLM system with its logic needs to be available to the user offline. This is ultimately limited by the user’s classical capabilities in downloading and then storing for later use. By contrast, the quantum-delayed decision scheme needs limited space for only one question and answer, albeit in quantum format. The quantum delayed decision model does not need to download the logic of *how* to obtain the answer of the question and therefore does not need to keep the logic of any language model. The quantum model needs basic manipulation of a small number of quantum bits. This makes the two approaches largely different in hardware, but also in the information that is tackled by the user and the size of the required memory.

The remainder of the paper is organized as follows. The next section provides a more detailed, formal description of Classical and Quantum Query Systems, thus providing the framework of this investigation. In Section 3, we derive the theoretical results supporting the idea of delayed decisions in the context of Binary Quantum Query Systems. These results are particularized in a few concrete examples experimentally validated using Qiskit, for up to 20 qubits, in Section 4. A generalization of our results to Non-Binary Query Systems is developed in Section 5. Finally, conclusions are presented in Section 6.

## 2. Classical vs. Quantum Query Systems

**Figure 1:**
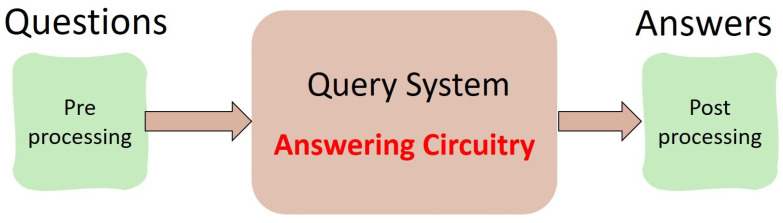
General schematics of a Query System modeled as a look-up table that accepts an index as input and produces the corresponding answer as output. For a user-friendly experience, there may be a pre-processing step in charge of refining the user query (formulated in a natural language) into an actual index in the look-up table and a post-processing phase in which the output is again translated (using natural language processing techniques) into a form that is easily understandable and appealing to the user.

A Classical Query System is, by definition, a service (usually provided by a server) through which users can interrogate or query the system in order to receive answers or information that is useful to the user, for example, to help with some decision-making processes. A concrete example of such systems is a Database Query System, in which users can ask for information related to a particular record in the database, or an AI agent that has been trained to provide answers to queries that are usually formulated in a specific domain of knowledge. For concreteness, we will henceforth assume that the Query System is modeled as a look-up table that accepts an index as input and produces the corresponding answer as output. Naturally, for a user-friendly experience, there may be a pre-processing step in charge of refining the user query (formulated in a natural language) into an actual index in the look-up table and a post-processing phase in which the output is again translated (using natural language processing techniques) into a form that is easily understandable and appealing to the user. Figure 1 depicts the general schematics of such a Query System.

The “question-to-encoder” dictionary is outside of the scope of this paper. In fact, this encoding is expected to be performed classically by a larger online entity, such as a server part of the Query System. Note that the online server provides the superposition to be stored by the user for later usage. Thus, the inner workings of the larger online entity could actually be an LLM or any other classical search engine.

A Quantum Query System (QQS) can be seen as an extension of a Classical Query System by harnessing the most important property of quantum information systems, namely *quantum parallelism*. Thus, besides being able to respond to single questions, just like a Classical System, a Quantum Query System can deal with multiple questions submitted simultaneously. As a trivial example, just to illustrate the concept, let us assume that a user may be interested in finding the answers to three questions: “Is the Internet fast?”, “Do I need to buy bread today?” and “How is my friend feeling?” If the user has access to a Classical Query System, then they have to submit three queries (one corresponding to each question) that the pre-processing stage may translate into three indices, e.g., 00, 10 and 11. Then the system may look up the answers and reply 1 (meaning “Fast”) for the first question, 0 (meaning “No”) to the second question and 0 (meaning “Happy”) to the last question. However, if the user has access to a Quantum Query System, then they can formulate a single query encompassing all three questions in the following way:

(1) $$|Query\rangle =\frac{1}{\sqrt{3}}(|00\rangle +|10\rangle +|11\rangle ).$$

When the Quantum Query System receives the above query as input, the answering circuitry produces a superposition in which each answer is appended to the corresponding question:

(2) $$|Answer\rangle =\frac{1}{\sqrt{3}}(|001\rangle +|100\rangle +|110\rangle ).$$

**Figure 2:**
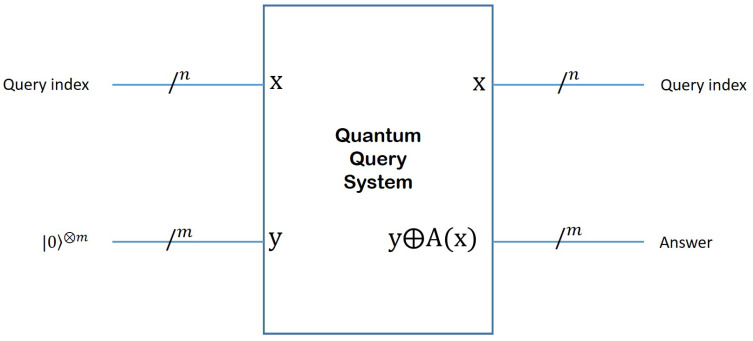
Inputs and outputs of a Quantum Query System. Since any quantum circuit has to be reversible, the inputs to the QQS have to be preserved as part of the output. Thus, the top *n* qubits (labeled as *x*), which encode the question index, appear unchanged at the output. The situation is different with the bottom *m* qubits, which are reserved for producing the answer A(x). The bitwise modulo2 operation between the bottom input *y* and A(x) is designed to maintain reversibility. By setting *y* to 0, the quantum circuitry of the QQS will produce the answer to question *x* (labeled A(x)) in the bottom *m* qubits of the output.

Since any quantum circuit has to be reversible, the inputs to the QQS have to be preserved as part of the output, as shown in Figure 2. Thus, the top *n* qubits (labeled as *x*), which encode the question index, appear unchanged at the output. The situation is different with the bottom *m* qubits, which are reserved for producing the answer A(x). The bitwise modulo2 operation between the bottom input *y* and A(x) is designed to maintain reversibility. By setting *y* to 0, the quantum circuitry of the QQS will produce the answer to question *x* (labeled A(x)) in the bottom *m* qubits of the output. In the case of the simple example described above, n=2 and m=1.

If the Query System is available without any interruptions and is guaranteed to service any user query at the moment the user needs an answer, then the Quantum System does not offer any advantage over a Classical one. However, quantum parallelism may help in situations where the user anticipates that access to the Query System will not be possible in the future, for example, because of the geographical location of the user at that point, due to a particular task that the user has to accomplish. The user can prepare in advance with all the information that will be needed by querying the system repeatedly until all required answers have been provided. If the number of queries is large, this may incur a high cost for the user. Therefore, in the following section, we will investigate the scenario in which the user submits a single query to the QQS, a query that encompasses all possible questions for which the user may need an answer in order to complete the future task.

As possible real-world use case scenarios for QQSs with delayed decisions, we mention a Satellite Environmental Monitoring System and a Privacy-Preserving Cloud Service. In the first case, imagine a satellite carrying a quantum processing unit that formulates multiple environmental queries in superposition (e.g., surface water quality, forest biomass index, atmospheric CO_2_ concentrations, etc.) over various regions of Earth. The data making up the answer to these queries coexist in a single quantum register during each orbit, even while the satellite is out of contact with any ground station. Because the satellite can only download a certain amount of data while it passes over a ground station, the ground controller decides which piece of information is most important during that particular pass of the satellite and instructs the quantum processor to selectively boost the amplitude of the desired data, before measuring the quantum register and sending down to Earth the measurement outcome. Similarly, in order to prevent a cloud server from tracking clients, thereby building a profile of the particular queries a client submits over time, the client can encode multiple possible searches in a single query and extract from the superposition of answers the one it actually has an interest in, without the service provider knowing exactly which particular search was ultimately resolved.

It is important to emphasize that, in most scenarios involving delayed decisions, at the moment of query submission, the user may be ignorant as to which particular piece of information (i.e., answer) will be needed to accomplish the task. The reply from the QQS will come in the form of a large superposition, similar to the one in Equation (2), with as many terms as the number of questions and the answer to each question appended to the corresponding question index. This quantum superposition state needs to be stored in a quantum register until the “decision moment”, when the relevant answer is to be extracted from the superposition of all answers. Therefore, the quantum model proposed in this paper needs quantum memories in order to work. Depending on the length of time that the quantum states keep their superposition before undergoing decoherence, the scope of a practical application of the scheme presented here has limitations. As of now, experiments show that the coherence of quantum states has been maintained for intervals ranging from 0.6 ms in 2016 [24] for optical qubits, to one hour as an atomic frequency in 2021 [25], to a 6 h spin coherence [26]. For our delayed decision model, the necessity of quantum memories is clear from the need to undergo several steps before retrieving the answer. From this perspective, our model shows a unique application of quantum memories, where the time of coherence directly affects how long the delay can stretch until a decision is made. The contribution of this paper strengthens the importance of continuing to research and extend the lifetime of a quantum ensemble in superposition.

We next examine the extent to which quantum information processing can help in order to extract the desired answer from the superposition of all answers at the (future) moment when the user knows exactly which particular answer is helpful for the task at hand.

## 3. Binary Quantum Query System with Delayed Decisions

For simplicity, let us assume that the Quantum Query System provides only binary answers (“Yes/No”, “Fast/Slow”, “Happy/Sad”, etc.), such that parameter *m* from Figure 2 is set to 1. The possible generalization to higher values will be discussed in Section 5. The value of *n*, on the other hand, is left unrestricted. Under these requirements, we first show in detail in what follows how Grover’s algorithm can be customized to our particular problem in order to boost the amplitude of the answer to the question of interest. Then, based on our quantum approach, we derive the probability of obtaining the answer to the desired question to be

(3) $$\begin{array}{c} {\left(\frac{1}{2}+\frac{1}{2\sqrt{2^{n}}}\right)}^{2}, \end{array}$$

compared with the $1/2^{n}$ probability that a classical system has to “guess” the correct question ahead of time.

### 3.1. A Detailed Quantum Framework for the Delayed Decision Problem

If the query state space spans *n* qubits, the user can formulate a query that encompasses $2^{n}$ different questions, encoded in the following superposition state:

(4) $$|Query\rangle =\sum_{x=0}^{2^{n}-1}\frac{1}{\sqrt{2^{n}}}|x\rangle .$$

When given the above state as input, the Quantum Query System will produce the following output:

(5) $$|Answer\rangle =\sum_{x=0}^{2^{n}-1}\frac{1}{\sqrt{2^{n}}}|x\rangle |A\left(x\right)\rangle ,$$

where A(x) represents the binary answer to the question with index *x*. Now, without loss of generality, suppose that after a certain time following the receipt of the answer state from the QQS, the user realizes that the useful answer that needs to be extracted from state |Answer〉 is the one to the question with index x=0. A direct measurement of state |Answer〉 in the computational basis has a small chance (only $1/2^{n}$) of revealing the sought-after A(0). Consequently, the amplitude of the target index 0 has to be increased relative to the amplitudes of the other terms in the superposition, through the use of Grover’s algorithm [16]. The steps of the algorithm, particularized for our specific problem, are given below as Algorithm 1.

The two operators used in every iteration of Algorithm 1 are U and D. Operator U acts only on the target states (the terms in the superposition that need to have their amplitudes increased) and rotates their phase by π radians. Operator D, on the other hand, acts on all basis states of the (n+1)-qubit ensemble to which it is applied and rotates all of them by π radians around the average amplitude of all basis states.
**Algorithm 1** Modified Grover’s algorithm to extract the desired answer.t←0|Ψ(0)〉=|Answer〉**while** t<T **do**    |Ψ(t+1)〉=DU|Ψ(t)〉    t←t+1**end while**Measure state |Ψ(T)〉 in the computational basis.

**Figure 3:**
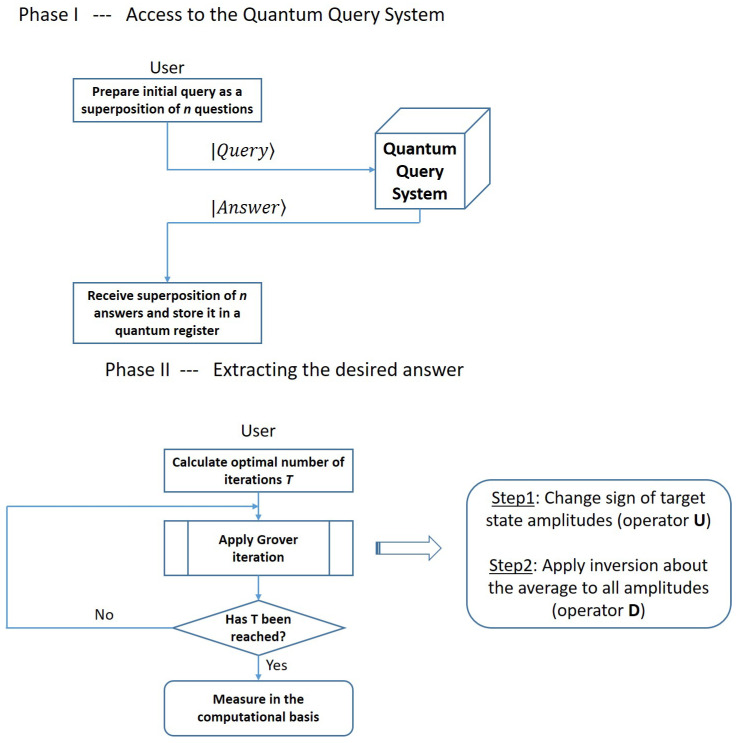
In Phase I, the user queries the system with a superposition of all questions that are deemed relevant to any decision taken in the near future and stores the superposition of answers received from the QQS in a quantum register. In Phase II, which takes place when the Quantum Query System is offline or inaccessible to the user, the iterations in Grover’s algorithm can be used to boost the amplitude of the desired term (the one encoding the answer to the relevant question for the decision to be made), such that the final measurement reveals the sought-after answer with good probability.

A flow chart presenting all steps of our approach in a visual form is shown in Figure 3. In Phase I, the user queries the system with a superposition of all questions that are deemed relevant to any decision taken in the near future and stores the superposition of answers received from the QQS in a quantum register. In Phase II, which takes place when the Quantum Query System is offline or inaccessible to the user, the iterations in Grover’s algorithm can be used to boost the amplitude of the desired term (the one encoding the answer to the relevant question for the decision to be made), such that the final measurement reveals the sought-after answer with good probability.

One crucial element that separates Algorithm 1 above from a typical application of Grover’s algorithm is the initial state |Ψ(0)〉. Usually, there is a *uniform* distribution of amplitudes over all basis states in the initial superposition, such that the target state(s) have the same initial amplitude as any of the other (non-target) basis states. Boyer et al. [27] showed that, starting from a uniform initial distribution of amplitudes, the optimal number of iterations after which the probability of measuring a target state is maximal is $T=O(\sqrt{N/r})$, where *r* is the number of target states from the total *N*.

However, the distribution of amplitudes in |Ψ(0)〉, which is the same state as |Answer〉 from Equation (5), is *not uniform*. The reason is simply because each of the questions |x〉, $x=0,\ldots ,2^{n}-1$ has only one answer, *either* |0〉 *or* |1〉, but not both. In other words, although |Ψ(0)〉 is a state vector in a $2^{n+1}$-dimensional space and should, therefore, be described by a linear combination of $2^{n+1}$ basis states, only half of them have non-zero amplitudes: the ones corresponding to actual answers. For example, if the answer to question |0〉 is 1 (i.e., A(0)=1), then the term |0〉|0〉 will not appear in the superposition state |Ψ(0)〉. Since, for each question index |x〉, only one answer is possible, the entire superposition |Ψ(0)〉 will consist of $2^{n}$ terms only, each with amplitude $1/\sqrt{2^{n}}$.

When acting on target states, operator U has to be designed such that it rotates both terms corresponding to a particular question index. Assuming again, for concreteness, that we are interested in retrieving A(0) from the superposition, U has to rotate phases of both the |0〉|0〉 term and the |0〉|1〉 term, since we do not know which one contains the actual answer. Therefore, matrix U is a diagonal matrix, where the first two elements on the main diagonal are −1 and the remaining elements on the main diagonal are 1:

(6) $$U_{ij}=\left\{\begin{array}{cc} -1 & ,\;\mathrm{if}\;i=j=0,1\\ 1 & ,\;\mathrm{if}\;i=j=2,3,\ldots ,2^{n+1}-1\\ 0 & ,\;\mathrm{if}\;i\neq j \end{array}\right.$$

The second operator D is the standard “inversion about the average” operator defined on n+1 qubits as follows:

(7) $$D_{ij}=\left\{\begin{array}{cc} \frac{1}{2^{n}}-1 & ,\;\mathrm{if}\;i=j\\ \frac{1}{2^{n}} & ,\;\mathrm{if}\;i\neq j \end{array}\right.$$

The key questions now are how many iterations *T* are needed in Algorithm 1 in order to amplify the amplitudes of the target states as much as possible and what is the probability of retrieving the desired information (i.e., A(0)) from the final measurement? As mentioned above, the analysis due to Boyer et al. [27] is not applicable, since the initial distribution of amplitudes is not uniform over all basis vectors. On the other hand, the case of arbitrary initial amplitudes in Grover’s algorithm was studied by Biron et al. [28]. They show that the optimal measurement time *T* is the same, asymptotically, in both scenarios (*uniform* distribution of amplitudes or *arbitrary* distribution) and is on the order of $\sqrt{N/r}$, where *r* is the number of target states and *N* is the total number of states. Furthermore, they find that *T* depends only on the initial average amplitudes of the target and non-target states.

Consequently, the time complexity of Algorithm 1 is $T=O\left(\sqrt{2^{n+1}/2}\right)=O\left(\sqrt{2^{n}}\right)$, where *n* represents the number of qubits used to encode the index of a question. In terms of the space complexity, since the algorithm employs *n* qubits for the question part and one qubit for the binary answer, there are n+1=O(n) qubits used in total. Note that these bounds are derived under the assumption of an ideal, error-free environment with perfect quantum operations. Any ancillary qubits required in a practical implementation for error-correcting purposes are not taken into consideration when stating the above complexities.

### 3.2. Probability of Success

In order to derive the probability of successfully retrieving the sought-after answer A(0) in the final measurement step of Algorithm 1, let us apply the results in [28] to our particular initial distribution of amplitudes in state |Ψ(0)〉. In our instance of Grover’s algorithm, the total number of states is $N=2^{n+1}$ and the number of target states r=2 (these are |0〉|0〉 and |0〉|1〉). The initial (at t=0) average of the amplitudes for the target states is the average of $1/\sqrt{2^{n}}$ and 0, since the question we are interested in has only one actual answer (labeled A(0)) and consequently, the state corresponding to the binary complement of A(0) will have amplitude 0 in the initial superposition |Ψ(0)〉. We denote this average of amplitudes of target states at moment t=0 as

(8) $$\bar{k}\left(0\right)=\frac{1}{2\sqrt{2^{n}}}.$$

Similarly, there are $2^{n+1}-2$ non-target states, only half of which have a non-zero amplitude in the initial state |Ψ(0)〉. Therefore, the initial average amplitude of the non-target states is

(9) $$\bar{l}\left(0\right)=\frac{1}{2^{n+1}-2}\cdot \frac{2^{n}-1}{\sqrt{2^{n}}}=\frac{1}{2\sqrt{2^{n}}},$$

which is the same as for the target states. Based on the ratio $\bar{k}\left(0\right)/\bar{l}\left(0\right)$, Biron et al. [28] have determined the optimal measurement time to be

(10) $$T=\frac{\frac{\pi }{2}-\arctan\left(\frac{\bar{k}\left(0\right)}{\bar{l}\left(0\right)}\sqrt{\frac{r}{N-r}}\right)}{\arccos(1-2\frac{r}{N})},$$

which, in our case, becomes

(11) $$T=\frac{\frac{\pi }{2}-\arctan\left(\sqrt{\frac{1}{2^{n}-1}}\right)}{\arccos(1-\frac{1}{2^{n-1}})}=O\left(\sqrt{2^{n}}\right).$$

To give a couple of concrete examples, if n=3, which means that the initial state |Ψ(0)〉 contains 8 non-zero terms corresponding to 8 questions and their answers, the value of *T* is approximately 1.67. It follows that two iterations of Algorithm 1 are enough to boost the amplitude of the target state to a maximum value before starting to decrease again if the algorithm is continued. If 10 qubits are used to encode a question index, then state |Ψ(0)〉 will span 1024 questions with corresponding answers, which requires T≈24.34 iterations in order for the amplitudes of the target states to reach their first maximum.

In their analysis, Biron et al. [28] also provide an upper bound on the probability of measuring a target state at the end of the algorithm after the optimal number of iterations *T* has been reached. This bound only depends on the variance of the initial amplitudes of the non-target states $\sigma_{l}^{2}$ and is given by Equation (12) below:

(12) $$P_{max}=1-\left(N-r\right)\sigma_{l}^{2}.$$

Recall that, for an arbitrary value of *n*, there are a total of $2^{n+1}-2$ non-target states, only half of which have a non-zero amplitude in the initial superposition |Ψ(0)〉. Consequently, in our case, the variance of the initial amplitudes of non-target states can be calculated as

(13) $$\sigma_{l}^{2}=\frac{1}{2^{n+1}-2}\left(\left(2^{n}-1\right){\left|\frac{1}{\sqrt{2^{n}}}-\frac{1}{2\sqrt{2^{n}}}\right|}^{2}+\left(2^{n}-1\right){\left|0-\frac{1}{2\sqrt{2^{n}}}\right|}^{2}\right)=\frac{1}{2^{n+2}}.$$

Substituting this in Equation (12), the maximum probability of measuring a target state becomes

(14) $$P_{max}=1-\left(2^{n+1}-2\right)\cdot \frac{1}{2^{n+2}}=\frac{1}{2}+\frac{1}{2^{n+1}}.$$

The first observation we can formulate about the result above is that the probability of seeing one of the target states through the final measurement can always be raised to more than 50%, regardless of the value of *n*, if we stop Algorithm 1 after an optimal number of iterations. However, this upper bound is not as good a result as it may look at first glance. And the reason is that we have *two* target states whose amplitudes are increased by the algorithm, one corresponding to the actual answer |0〉|A(0)〉 and the second one corresponding to the bit complement of the actual answer $\left|0\rangle \right|\bar{A(0)}\rangle$. Consequently, it is crucial to see how much each of these two target states is amplified in the end, such that when the final measurement is performed, we obtain the actual answer and not its complement. We do expect that the term |0〉|A(0)〉 will have a higher probability of being measured compared with $\left|0\rangle \right|\bar{A(0)}\rangle$, since the latter starts with a zero amplitude, but the subsequent calculations will make things more precise.

Let us denote by $\alpha =k_{1}\left(T\right)$ the amplitude of the term |0〉|A(0)〉 (the term we are interested in), as it appears in the state |Ψ(T)〉, at the end of Algorithm 1. Similarly, $\beta =k_{2}\left(T\right)$ is the amplitude of the term $\left|0\rangle \right|\bar{A(0)}\rangle$ (the term carrying the bit complement of the answer) in the same superposition state |Ψ(T)〉. At any time *t* during the execution of Algorithm 1, the amplitude of a target state $k_{i}\left(t\right)$ can be expressed based on the average amplitude of all target states at that moment $\bar{k}\left(t\right)$:

(15) $$k_{i}\left(t\right)=\bar{k}\left(t\right)+\Delta k_{i}\left(t\right).$$

As the analysis in [28] shows, the deviation from the average for a particular target state *i*, labeled as $\Delta k_{i}\left(t\right)$ in the equation above, is a time-independent quantity, meaning that it remains constant throughout the execution of Algorithm 1. Consequently, we can determine $\Delta k_{1}$ and $\Delta k_{2}$ for our two target states, based on the information we have at the moment t=0:

(16) $$\begin{array}{c} \Delta k_{1}=k_{1}\left(0\right)-\bar{k}\left(0\right)=\frac{1}{\sqrt{2^{n}}}-\frac{1}{2\sqrt{2^{n}}}=\frac{1}{2\sqrt{2^{n}}},\\ \Delta k_{2}=k_{2}\left(0\right)-\bar{k}\left(0\right)=0-\frac{1}{2\sqrt{2^{n}}}=-\frac{1}{2\sqrt{2^{n}}}. \end{array}$$

Based on the calculated values for $\Delta k_{1}$ and $\Delta k_{2}$, we can now express the amplitudes of the target states at time t=T as follows:

(17) $$\begin{array}{c} \alpha =k_{1}\left(T\right)=\bar{k}\left(T\right)+\Delta k_{1}=\bar{k}\left(T\right)+\frac{1}{2\sqrt{2^{n}}},\\ \beta =k_{2}\left(T\right)=\bar{k}\left(T\right)+\Delta k_{2}=\bar{k}\left(T\right)-\frac{1}{2\sqrt{2^{n}}}. \end{array}$$

Subtracting the two equalities above gives us the first equation directly relating α and β:

(18) $$\alpha -\beta =\frac{1}{\sqrt{2^{n}}}.$$

A second relation can be obtained from Equation (14), expressing the maximum probability of measuring a target state. Since $P_{max}$ is attained at t=T and the amplitudes of the two target states at that moment are α and β, respectively, it follows that

(19) $$P_{max}={\left|\alpha \right|}^{2}+{\left|\beta \right|}^{2}=\frac{1}{2}+\frac{1}{2^{n+1}}.$$

Equations (18) and (19) yield the following two possible sets of solutions for α and β:

(20) $$\begin{array}{c} \alpha =\frac{1}{2}+\frac{1}{2\sqrt{2^{n}}}\\ \beta =\frac{1}{2}-\frac{1}{2\sqrt{2^{n}}}, \end{array}$$

respectively,

(21) $$\begin{array}{c} \alpha =-\frac{1}{2}+\frac{1}{2\sqrt{2^{n}}}\\ \beta =-\frac{1}{2}-\frac{1}{2\sqrt{2^{n}}}. \end{array}$$

The dual set of solutions for α and β reflects the cyclical nature of Grover’s algorithm and, implicitly, that of our customized version. The amplitudes of the target states are amplified by each iteration in Algorithm 1 until they reach a point where the probability of measuring one of them is maximum. This optimal moment for measuring the superposition state is labeled as *T* and its formula is given in Equation (11). The values of α and β at moment t=T are given in the first set of solutions (Equation (20)). We note that, at this point, both α and β are positive and α>β, which means that we have a higher chance of obtaining the actual answer A(0) than its complement $\bar{A(0)}$ from the final measurement.

However, if the algorithm is not stopped at time t=T and we continue applying its iterations, then α and β will start decreasing, become negative, and reach a point where they are big enough in absolute value in order for the probability $P_{max}$ to be reached again. This moment corresponds to the second set of solutions (Equation (21)). However, at this point, |α|<|β|, and therefore, the probability of seeing $\bar{A(0)}$ instead of A(0) is higher. This periodic behavior—where the amplitudes α and β evolve continuously between the values in Equation (20) and those in Equation (21)—is exhibited for as long as the iterations in Algorithm 1 are unfolding. For concreteness, we next analyze the results obtained for some particular values of *n*.

## 4. A Few Concrete Examples

Consider first the case where n=3. We already mentioned in the previous section that in this case, Algorithm 1 acts on a four-qubit space, three of which encode the question index and the fourth one stores the answer. Therefore, the initial superposition |Ψ(0)〉 contains 8 non-zero terms corresponding to 8 questions with their answers. According to the calculations, the optimal moment to measure the superposition state is at time t=T≈1.67. At this time, the probabilities of measuring the answer bit A(0), and subsequently its complement $\bar{A(0)}$, are bounded by

(22) $$\begin{array}{c} {\left|\alpha \right|}^{2}={\left(\frac{1}{2}+\frac{1}{2\sqrt{2^{3}}}\right)}^{2}\approx 0.46\\ {\left|\beta \right|}^{2}={\left(\frac{1}{2}-\frac{1}{2\sqrt{2^{3}}}\right)}^{2}\approx 0.1 \end{array}$$

Since we cannot execute fractions of iterations, the best we can do is stop the algorithm after two iterations. Applying the operators U and D on the initial state |Ψ(0)〉 two times will boost the values of α and β to $15\sqrt{2}/32$ and $7\sqrt{2}/32$, respectively. This corresponds to a probability of measuring A(0) of approximately 0.44 and a 0.096 probability of obtaining $\bar{A(0)}$ in the final measurement. These values are very close to the bounds obtained in Equation (22).

Continuing the iterations of Algorithm 1, α and β begin to decrease and at time t=6 (i.e., after six iterations), their values are very close to the theoretical bounds derived in Equation (21). More precisely, α≈−0.32 and β≈−0.68, giving a probability of about 0.1 to measure A(0) and a 0.458 probability to observe $\bar{A(0)}$ in the measurement. We note that the probabilities of obtaining A(0) and subsequently $\bar{A(0)}$ have effectively swapped, compared with the situation after two iterations. Nevertheless, the two probabilities combined are again very close to the maximum theoretical probability of

(23) $$P_{max}=\frac{1}{2}+\frac{1}{2^{4}}=0.5625,$$

just as it happened at moment t=2. This cycle of approximately four iterations between the moments when the probability of measuring a target state is maximum may continue indefinitely. In our particular case, the next recurrence occurs after 10 iterations, when the amplitudes of A(0) and $\bar{A(0)}$ are again positive and close to the bounds calculated in Equation (22). In general, the exact moments when the amplitudes of α and β are at a maximum (in absolute value) is given by the following formula:

(24) $$T=\frac{\left(k+\frac{1}{2}\right)\pi -\arctan\left(\sqrt{\frac{1}{2^{n}-1}}\right)}{\arccos(1-\frac{1}{2^{n-1}})},\;\mathrm{for}\;k=0,1,2,\ldots .$$

If n=3, taking *k* to be 0,1, and 2 yields the following approximate values for *T*: 1.67, 6.02, and 10.36, respectively. Given the fact that, among these three values, the middle one is closest to an integer, it is not surprising that the best overall probability to measure a target state is achieved after six iterations. Consequently, if we decide to stop the algorithm after six iterations instead of two, in order to take advantage of the best possible probabilities, we just need to remember that the most likely outcome is the opposite of the actual answer and interpret the result obtained accordingly.

Increasing the value of parameter *n* allows us to store a significantly larger number of questions and their answers in the initial state |Ψ(0)〉. On the other hand, the higher the value of *n*, the smaller the difference between α and β becomes, as can be seen from Equation (18). This means that the probabilities of obtaining A(0) and $\bar{A(0)}$ at the end of Algorithm 1 will be closer together as the number of qubits increases. Therefore, after a certain threshold, the results obtained will become statistically irrelevant, as very little information about the sought-after answer can be extracted through the final measurement.

In the case of n=10, for example, the quantum register on which Algorithm 1 is applied consists of 11 qubits (including the answer qubit) and can therefore hold up to 1024 different question–answer pairs. However, the confidence that the measurement at the end yields the actual answer to the question of our choice (and not its complement) also reduces significantly. According to Equation (20), the probability of retrieving A(0) is approximately 0.266, while the probability of seeing $\bar{A(0)}$ after the measurement is a little lower, at 0.235. These probabilities correspond to the moment when $P_{max}$ is reached, which, for n=10, happens for the first time at T≈24.3.

### 4.1. Experimental Simulations

The theoretical results calculated above have been confirmed by practical experiments conducted on a quantum simulator using Qiskit. W conducted five rounds of experiments for n=3, n=5, n=8, n=16, and n=20. This means that the correct answer has to be retrieved from a superposition of 8, 32, 256, 65,536, and 1,048,576 questions, respectively. Since the answer is binary (encoded on a single qubit), Algorithm 1 acts on a vector space spanned by 4, 6, 9, 17, and 21 qubits, respectively. In all experiments, we seek to retrieve the answer to the question with index 0, which is set to 0 in the initial superposition (A(0)=0). The reason for stopping at 21 qubits is twofold. Firstly, it is clear from the theoretical analysis that the difference between the probability of obtaining the correct answer and the probability of obtaining the bit complement of the correct answer all but vanishes for larger values of *n*. Secondly, the simulator itself cannot deal with a higher number of qubits and runs out of memory, since the number of iterations in the modified Grover algorithm becomes too computationally intensive.

**Figure 4:**
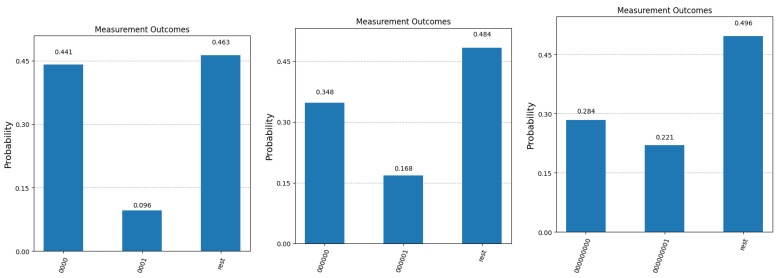
Experimental results after 100,000 runs of the simulator for n=3, n=5, and n=8. The first bar in each graph represents the probability of obtaining the corect answer to the question with index 0 (labeled as A(0)). The second bar in each graph shows the probability of measuring the binary complement of A(0). The third bar depicts the cumulative probability of fishing out any of the other questions that are part of the superposition state at the end of Algorithm 1.

**Figure 5:**
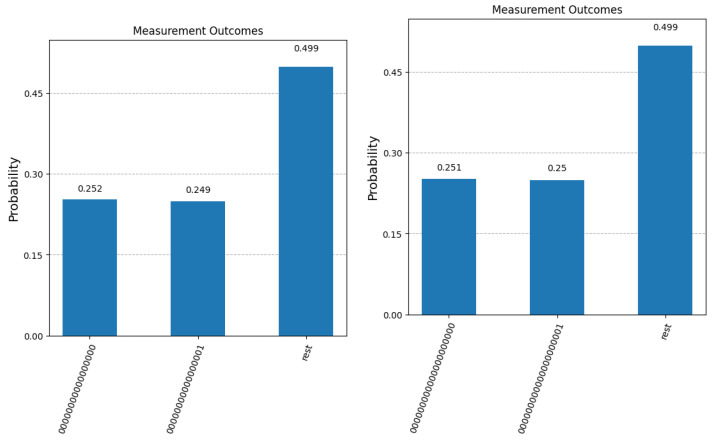
Experimental results after 100,000 runs of the simulator for n=16 and n=20. The first bar in each graph represents the probability of obtaining the correct answer to the question with index 0 (labeled as A(0)). The second bar in each graph shows the probability of measuring the binary complement of A(0). The third bar depicts the cumulative probability of fishing out any of the other questions that are part of the superposition state at the end of Algorithm 1.

For all five values of *n*, we present the final measurement statistics as probabilities of obtaining term |0〉|0〉 (correct answer), |0〉|1〉 (binary complement of the correct answer) and any other term in the superposition (corresponding to the case where Algorithm 1 fails to fish out the desired question). Since the quantum simulator only returns the number of counts for each possible measurement outcome, we computed the probabilities by dividing each count value to 100,000 (the total number of times each experiment was repeated). The results are presented in Figure 4 and Figure 5. We mention here that the quantum simulator assumes an ideal, error-free environment, where all operations involved—from the preparation of the initial state—all of the applied quantum gates, and the measurement operation at the end is not affected by noise, decoherence or any other errors. The optimal number of Grover iterations required to obtain the results in Figure 4 and Figure 5 are T=2 for n=3, T=4 for n=5, T=12 for n=8, T=201 for n=16, and finally, T=804 for n=20.

**Figure 6:**
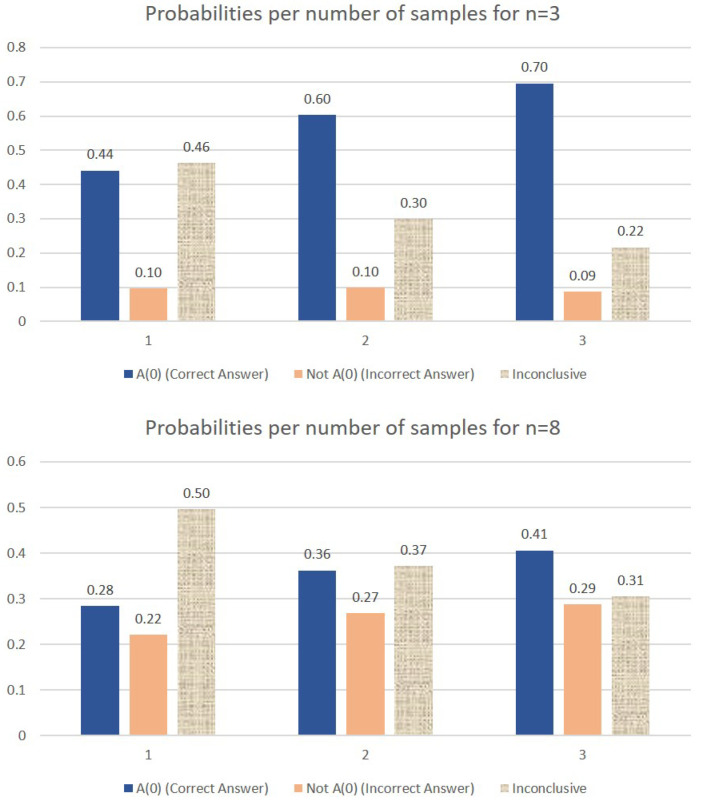
Comparative probabilities of obtaining the correct answer if one, two, or three samples of the initial query response are available, in the case of n=3 and n=8. When two samples are available, the probability of obtaining the correct answer A(0) includes the case where both samples are measured as |0〉|0〉 as well as the cases where one measurement yields |0〉|0〉 and the other one fails to retrieve the desired question index. Similarly, the third bar in each graph (labeled as “Inconclusive”) includes the case where both measurements fail to fish out the desired question index as well as the situation where one measurement yields A(0) (the correct answer) and the other measurement yields $\bar{A(0)}$ (the binary complement of the correct answer). For the cases where three samples are available, a successful measurement (first bar) includes the following scenarios: at least two measurements yield |0〉|0〉 or, only one measurement give the correct answer and the other two fail to retrieve the correct question index.

We first note that in the case of n=3, the experimental values observed for |0000〉 and |0001〉 match the probabilities of measuring A(0) (0.44) and $\bar{A(0)}$ (0.096) calculated in the previous section. Secondly, as expected, when the number of qubits increases, it becomes more difficult to separate the correct answer A(0) (first bar in all five graphs) from the incorrect answer $\bar{A(0)}$ (second bar in all graphs). This separation can be improved if we relax the constraint that only one query to the system is allowed. Figure 6 shows the improvement in the confidence that the result of the measurement is the sought-after answer if more than one copy of the initial superposition state (the response from the Query System) is available.

Note that in the case where two samples are available, the probability of obtaining the correct answer A(0) includes the case where both samples are measured as |0〉|0〉 as well as the cases where one measurement yields |0〉|0〉 and the other one fails to retrieve the desired question index. Similarly, the third bar in the graph (labeled as “Inconclusive”) includes the case where both measurements fail to fish out the desired question index as well as the situation where one measurement yields A(0) (the correct answer) and the other measurement yields $\bar{A(0)}$ (the binary complement of the correct answer).

For the cases where three samples are available, a successful measurement (first bar) includes the following scenarios: at least two measurements yield |0〉|0〉 or, only one measurement give the correct answer and the other two fail to retrieve the correct question index. Again, we notice that the improvement in the separation between the first two bars in each graph decreases with the value of *n*, but increases with the number of samples.

A general theoretical formula describing the expected probability of successfully measuring the |0〉|A(0)〉 component, if *k* samples are available, is given below:

(25) $$P_{success}=\sum_{i=1}^{k}C\left(k,i\right)p^{i}\sum_{j=0}^{min(i-1,k-i)}{\left(1-p-q\right)}^{k-i-j}C\left(k-i,j\right)q^{j}.$$

In the above formula, variable $p={\left|\alpha \right|}^{2}$ denotes the probability of obtaining |0〉|A(0)〉 in the final measurement step of Algorithm 1, variable $q={\left|\beta \right|}^{2}$ denotes the probability of measuring $\left|0\rangle \right|\bar{A(0)}\rangle$, and consequently, 1−p−q represents the cumulative probability of fishing out any of the other terms in the superposition. The formula is derived based on a simple majority approach, meaning that, in order for the procedure to be considered successful, more measurements (out of the total *k*) have to collapse onto the correct answer |0〉|A(0)〉 than onto its the binary complement $\left|0\rangle \right|\bar{A(0)}\rangle$.

For example, if two samples of the initial superposition |Ψ(0)〉 received from the QQS are available (that is, k=2), then Equation (25) becomes

(26) $$\begin{array}{cc} P_{success} & =C\left(2,1\right)p^{1}{\left(1-p-q\right)}^{1}C\left(2,0\right)q^{0}+C\left(2,2\right)p^{2}{\left(1-p-q\right)}^{0}C\left(0,0\right)q^{0}\\ \; & =2p\left(1-p-q\right)+p^{2}. \end{array}$$

Indeed, with two measurements, the procedure is considered successful if any of the two measurements retrieve |0〉|A(0)〉 (probability *p*) and the other one fails to retrieve the sought-after question–answer pair (probability 1−p−q), or both measurements are successful (term $p^{2}$ in the above formula). In general, increasing the value of *k* leads to higher success probabilities (as one would expect), but at the same time reduces the advantage of the quantum approach over the classical approach, since, when more queries are allowed to be sent to the Query System, the classical success probability increases as well.

The particular cases analyzed in this section suggest that quantum computation techniques can indeed support delayed-decision scenarios within certain parameters. The main issue is finding the optimal trade-off between the capacity of the quantum register (i.e., the number of question–answer pairs that can be stored in advance in the quantum register through a superposition state) and the confidence of the final measurement to yield the actual answer to the question chosen by the user at a later time. The values obtained above tend to indicate that a quantum register with less than a dozen qubits is still capable of storing hundreds of answers to different queries, and at the moment when a particular answer is needed, Algorithm 1 can provide that answer with a good probability. Furthermore, this probability can always be increased (sometimes substantially), if two or three copies of the quantum register are available. Naturally, this assumes that the user has queried the QQS two or three times with the same superposition of questions.

We close this section devoted to experimental validations of delayed decisions in Quantum Query Systems by addressing the issue of quantum errors affecting the operations in Algorithm 1. Any quantum machine in existence today struggles with the plague of quantum errors affecting their operations. The particular type of errors occurring during a certain computation depends entirely on the quantum hardware or the engineering details through which a quantum computer chooses to implement a qubit (in other words, the physical embodiment of the qubit). The error rate in quantum gates seems to be the major obstacle towards scalable quantum computing, but the technology is still in its infancy and future advances will probably improve this aspect significantly.

Since Grover’s algorithm is a powerful tool with broad applicability to any generic unstructured database search, researchers have also tackled the issue of quantum errors impeding its operation [29,30,31,32,33]. A common conclusion revealed by these studies is that the error approximately grows with the inverse of the search space. Recent advances aim to develop variants of Grover’s algorithm that are more resilient to noise. Leng et al. [30] claim exponential improvement on error threshold. Ishii et al. [31] investigates how coherent errors stemming from control imprecision impact Grover’s algorithm in a trapped ion device. They demonstrate how combining randomized compiling with error detection significantly reduces errors in circuits with up to 10 qubits. Similarly, the study by Kumar et al. [33] shows that applying measurement error mitigation improves accuracy, especially in small-scale implementations. Our theoretical analysis reinforced by the practical experiments have shown that the technique of Delayed Decisions offers the best chances of success for a small number of qubits (less than a dozen), but that can still allow dozens or hundreds of questions to be encoded in a single query. The fact that it is easier for implementations of Grover’s algorithm using fewer qubits to maintain the accuracy of the computation works in favor of our proposed technique for Delayed Decisions.

**Figure 7:**
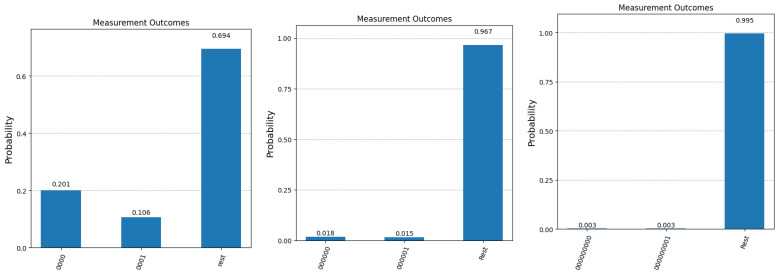
Experimental results after 8192 runs on an actual IBM quantum machine for n=3, n=5, and n=8. The first bar in each graph represents the probability of obtaining the correct answer to the question with index 0 (labeled as A(0)). The second bar in each graph shows the probability of measuring the binary complement of A(0). The third bar depicts the cumulative probability of fishing out any of the other questions that are part of the superposition state at the end of Algorithm 1.

To illustrate the impact of quantum errors on the steps of our modified Grover algorithm, we ran a few experiments on one of the actual quantum machines that are available on the IBM Quantum Platform. Figure 7 presents the measurement statistics in the case of 4 qubits (n=3), 6 qubits (n=5) and 9 qubits (n=8), respectively. All experiments were run for 8192 shots, the maximum number allowed by the IBM on their quantum machines. As expected, the results that are closer to the simulator were obtained for the smaller number of qubits (4). For a higher number of qubits, the number of iterations required to retrieve the correct answer increases, leading to an accumulation of errors. This can be seen in the last two graphs, where the number of terms in the superposition also increases significantly (64 terms for n=5 and 512 terms for n=8), resulting in the leveling of amplitudes across all terms in the superposition. In the first graph, we still have a meaningful difference between the two bars depicted; this difference is drastically reduced in the middle graph and disappears completely in the third one. These experiments accurately reflect the current status of practical quantum computing, where scalability remains the main issue. Nevertheless, we remain confident that future developments in quantum hardware will significantly improve this crucial aspect.

### 4.2. Acting on a Smaller Subspace

Throughout our investigation so far, the quantum operators in Algorithm 1 always act on the entire Hilbert space spanned by the n+1 qubits encoding the question index and its binary answer. But because each question |i〉 can only have one answer (either 0 or 1), the initial state |Ψ(0)〉 is a superposition of $2^{n}$ terms (from the possible $2^{n+1}$), with the other half having a zero amplitude. Since we do not know exactly which terms are present in the initial superposition and which are not (we do not know the answers to any of the questions), an alternative idea would be to act only on the *n* qubits that make up the question index.

From the point of view of Algorithm 1, this means that operators U and D can be decomposed into a tensor product between a $2^{n}\times 2^{n}$ operator acting on the *n* qubits representing the question index and the 2×2 identity operator acting on the answer qubit. The phase shift operator U is not affected by the fact that we now act only on *n* qubits instead of all n+1. Assuming again, for concreteness, that we want to extract A(0) from the initial superposition state, the $2^{n}\times 2^{n}$ operator that rotates the phase of basis vector |0…0〉 tensored with the identity operator coincides with the $2^{n+1}\times 2^{n+1}$ operator that flips the sign of both |0…0〉|0〉 and |0…0〉|1〉 basis vectors:

(27) $$\left(\begin{array}{ccccc} -1\; & 0\; & 0\; & \ldots & 0\\ 0\; & 1\; & 0\; & \ldots & 0\\ 0\; & 0\; & 1\; & \ldots & 0\\ \vdots \\ 0\; & 0\; & 0\; & \ldots & 1 \end{array}\right)⊗\left(\begin{array}{c} 1\;0\\ 0\;1 \end{array}\right)=\left(\begin{array}{ccccc} -1\; & 0\; & 0\; & \ldots & 0\\ 0\; & -1\; & 0\; & \ldots & 0\\ 0\; & 0\; & 1\; & \ldots & 0\\ \vdots \\ 0\; & 0\; & 0\; & \ldots & 1 \end{array}\right)$$

However, the situation is different with the “inversion about the average” operator D: the n+1-qubit version, given in Equation (7), is different from the *n*-qubit version tensored with identity:

(28) $$\left(\begin{array}{ccccc} \frac{2}{2^{n}}-1\; & \frac{2}{2^{n}}\; & \frac{2}{2^{n}}\; & \ldots & \frac{2}{2^{n}}\\ \frac{2}{2^{n}}\; & \frac{2}{2^{n}}-1\; & \frac{2}{2^{n}}\; & \ldots & \frac{2}{2^{n}}\\ \frac{2}{2^{n}}\; & \frac{2}{2^{n}}\; & \frac{2}{2^{n}}-1\; & \ldots & \frac{2}{2^{n}}\\ \vdots \\ \frac{2}{2^{n}}\; & \frac{2}{2^{n}}\; & \frac{2}{2^{n}}\; & \ldots & \frac{2}{2^{n}}-1 \end{array}\right)⊗\left(\begin{array}{c} 1\;0\\ 0\;1 \end{array}\right)=\left(\begin{array}{ccccccc} \frac{2}{2^{n}}-1\; & 0\; & \frac{2}{2^{n}}\; & 0\; & \ldots & \frac{2}{2^{n}}\; & 0\\ 0\; & \frac{2}{2^{n}}-1\; & 0\; & \frac{2}{2^{n}}\; & \ldots & 0\; & \frac{2}{2^{n}}\\ \frac{2}{2^{n}}\; & 0\; & \frac{2}{2^{n}}-1\; & 0\; & \ldots & \frac{2}{2^{n}}\; & 0\\ \vdots \\ 0\; & \frac{2}{2^{n}}\; & 0\; & \frac{2}{2^{n}}\; & \ldots & 0\; & \frac{2}{2^{n}}-1 \end{array}\right)$$

Since half of the elements in the matrix above are 0, the resulting operator is generally less effective at amplifying the amplitude of the target state, compared with the operator D employed in Algorithm 1, especially if the number of iterations required is not very small. In addition, operator D from Algorithm 1 always produces the same results, regardless of which answers are 0 and which are 1, while the performance of the operator above is influenced by which terms appear in the initial superposition state. To exemplify, suppose that the initial state on which Algorithm 1 acts upon is a superposition of eight question–answer pairs as follows:

(29) $$|\Psi \left(0\right)\rangle =\frac{1}{2\sqrt{2}}(|0\rangle |1\rangle +|1\rangle |0\rangle +|2\rangle |0\rangle +|3\rangle |1\rangle +|4\rangle |0\rangle +|5\rangle |1\rangle +|6\rangle |0\rangle +\left|7\rangle |1\rangle \right)$$

Acting on all four qubits with Algorithm 1 will increase the amplitude of term |0〉|1〉 from the initial value of $1/2\sqrt{2}$ to $15/16\sqrt{2}$ in just two iterations. On the other hand, if we act only on the first three qubits (representing the question index), then after two iterations, the amplitude of term |0〉|1〉 reaches only $10/16\sqrt{2}$.

## 5. Non-Binary Quantum Query Systems

We conclude our investigation into Quantum Query Systems and the extent to which they can be used to support delayed decisions by addressing the issue of whether a QQS can produce non-binary answers as responses to questions. In other words, we next examine the generalization to more than one qubit being used to encode the answer to a question. The first step in this direction would be to assume that the answer to any question is a vector living in a four-dimensional space spanned by two qubits. If we still consider that an *n*-qubit register is used to encode the question index, then we reach a total number of states of $N=2^{n+2}$ from which r=4 is the target state, since there are now four possible answers to a question.

As it turns out, the average of amplitudes of target states at time t=0 is still equal to the initial average amplitudes of the non-target states:

(30) $$\bar{k}\left(0\right)=\bar{l}\left(0\right)=\frac{1}{4\sqrt{2^{n}}}.$$

Consequently, the optimal measurement time (i.e., the number of iterations of Grover’s algorithm that maximizes the probability of picking up a target state) is still $O(\sqrt{2^{n}})$, unchanged from the case of binary answers, with the exact formula given in Equation (11). Furthermore, based on the variance of the initial amplitudes of non-target states,

(31) $$\begin{array}{cc} \sigma_{l}^{2} & =\frac{1}{2^{n+2}-4}\left(\frac{2^{n+2}-4}{4}{\left|\frac{1}{\sqrt{2^{n}}}-\frac{1}{4\sqrt{2^{n}}}\right|}^{2}+\frac{(2^{n+2}-4)\cdot 3}{4}{\left|0-\frac{1}{4\sqrt{2^{n}}}\right|}^{2}\right)\\ \; & =\frac{3}{16\cdot 2^{n}}, \end{array}$$

where the upper bound on the probability of measuring a target state after an optimal number (*T*) of iterations of performing Grover’s algorithm is

(32) $$P_{max}=1-\left(N-r\right)\sigma_{l}^{2}=1-\left(2^{n+2}-4\right)\cdot \frac{3}{16\cdot 2^{n}}=\frac{1}{4}+\frac{3}{4\cdot 2^{n}}.$$

According to this result, the chance of obtaining one of the target states in the final measurement is always greater than 25%, with significantly larger values being possible for small values of *n*, which means a relatively small number of questions $2^{n}$ that can be asked simultaneously. The result above can easily be generalized to an arbitrary number *k* of qubits used by a Quantum Query System to encode the answer to a question:

(33) $$P_{max}=\frac{1}{2^{k}}+\frac{2^{k}-1}{2^{n+k}}.$$

As expected, the more qubits are used to detail the response to a question, the more difficult it is to increase the magnitude of the target states in the increasingly larger superposition returned by the Quantum Query System. Therefore, in practice, the number of qubits used to encode answers to questions should be kept to a minimum, unless multiple copies of the initial superposition state obtained from the QQS are available through multiple interrogations.

## 6. Conclusions

Quantum superposition of states is the key quantum mechanical property allowing quantum algorithms to outperform their classical counterparts or endowing quantum cryptographic protocols with levels of security that are unattainable for protocols implemented based on the laws of classical physics. In this manuscript, we have investigated the potential advantages that a quantum mechanical implementation can bring to the field of generic Query Systems by harnessing the massive parallelism implicit in quantum superpositions.

Our study has revealed that Quantum Query Systems have the advantage over classical systems in situations where the number of queries is severely limited and continuous access to the server or oracle is not always possible. Under these adverse conditions, a quantum strategy can be formulated to take advantage of quantum parallelism and extract the desired information from a pre-stored superposition at a time when querying the system is not possible. The strategy works best with binary answers, but may be extended to answers encoded into several qubits if we also allow for several queries to be sent to the oracle. Additionally, our investigation has revealed that the best strategy for successfully retrieving the desired answer from the superposition of question–answer pairs is to apply Grover’s algorithm on the entire Hilbert space where the superposition is defined, and not just on the subspace spanned by the qubits encoding the question indices (without acting on the answer qubits).

The quantum simulations performed for various sizes up to 20 qubits validate the theoretical analysis performed in order to derive the probability of success for our proposed quantum approach if we assume an ideal, error-free environment. As expected, experiments run on an actual IBM quantum machine, where quantum gates are affected by noise and decoherence, show a deviation from the theoretical results and the ideal simulations. The higher the number of qubits, the more Grover iterations are required to retrieve the correct answer, leading to an accumulation of errors. The net effect is a leveling of amplitudes across all terms in the superposition, which reduces the probability of successfully extracting the desired answer. Nevertheless, quantum technologies are still in their infancy and we remain confident that current research efforts will significantly improve the accuracy of quantum computations.

## Data Availability

The original contributions presented in this study are included in the article/Supplementary Materials. Further inquiries can be directed to the corresponding author.

## Supplementary Materials

The code used in the experimental simulations conducted in Section 4.1 is available at https://github.com/madi12c/Quantum-Delayed-Decision-Qiskit-Experiments/ (accessed on 20 August 2025).

## Abbreviations

The following abbreviations are used in this manuscript:

AI	Artificial Intelligence
ERP	Enterprise Resource Planning
GQL	Graph Query Language
QAOA	Quantum Approximate Optimization Algorithm
QQL	Quantum Query Language
QQS	Quantum Query System
LLM	Large Language Model
SQL	Structured Query Language
